# Mesenchymal stem cell-derived small extracellular vesicles suppress pyroptosis by delivering miR-125a-5p to improve acute kidney injury in sepsis

**DOI:** 10.1038/s41420-026-03143-6

**Published:** 2026-05-19

**Authors:** Feng Chen, Tao-Tao Tang, Zhi-Qing Chen, Qin Yang, Yue Zhang, Yi-Lin Zhang, Jing Song, Meng-Yun Wang, Hong-Bin Yang, Min Yang, Suo-Fu Qin, Zhe Guo, Xue-Song Wang, Zhong Wang, Lin-Li Lv, Bi-Cheng Liu

**Affiliations:** 1 Department of Nephrology, Zhongda Hospital, Southeast University School of Medicine, Nanjing, China; 2 School of Clinical Medicine, Tsinghua University, Beijing, China; 3 Shenzhen Kexing Pharmaceutical Co. Ltd., Shenzhen, China

**Keywords:** Necroptosis, Mechanisms of disease, RNAi, Transcriptomics, Mesenchymal stem cells

## Abstract

Sepsis-induced acute kidney injury (S-AKI) is a life-threatening condition driven by excessive immune inflammation, and effective treatments remain lacking. Mesenchymal stem cell-derived small extracellular vesicles (MSC-sEV) have been demonstrated to possess potent immunomodulatory activity. This study aimed to investigate the role and underlying mechanism of MSC-sEV in S-AKI. We established in vivo and in vitro models of S-AKI and employed techniques such as small RNA sequencing, transcriptome sequencing, luciferase reporter assays, and engineered gene editing to validate therapeutic efficacy and elucidate mechanisms. Results demonstrated that in S-AKI, MSC-sEV homed to injured kidneys and were internalized by renal tubular epithelial cells, significantly ameliorating renal damage and improving survival rates. Mechanistically, MSC-sEV delivered miR-125a-5p to target and inhibit TNFR2 expression, thereby blocking TNF-driven pyroptosis mediated by the NF-κB/NLRP3 signaling pathway. Furthermore, engineered modification with the EXOMotif GGAG significantly enhanced MSC-sEV delivery of miR-125a-5p and inhibition of TNFR2. In conclusion, this study demonstrates that MSC-sEV represent a promising drug delivery vehicle with substantial targeted therapeutic potential for S-AKI.

## Introduction

Sepsis is defined as a life-threatening multiorgan dysfunction caused by a dysregulated host response to infection, affecting approximately 50 million people worldwide annually with a mortality rate of 20–30% [1–3]. Sepsis-induced acute kidney injury (S-AKI) occurs in up to 60% of septic patients and accounts for 45–70% of AKI cases in intensive care units [4]. Alarmingly, S-AKI increases in-hospital mortality by 6- to 8-fold compared to non-septic AKI and elevates the risk of chronic kidney disease (CKD) in survivors by threefold [5]. Despite its clinical significance, current management remains limited to supportive care, highlighting an urgent need for specific therapies.

The pathogenesis of S-AKI involves complex interactions between systemic inflammation and renal cellular injury [6]. Renal tubular epithelial cells (RTECs), with their high metabolic activity, are especially susceptible to sepsis-induced ischemia, oxidative stress, and inflammatory damage [7]. During S-AKI, an early cytokine storm triggers microcirculatory dysfunction and exacerbates RTEC injury, creating a vicious cycle that drives both acute renal impairment and long-term progression to CKD [8, 9]. These observations suggest that early intervention targeting RTEC injury and inflammatory cascades could represent a promising therapeutic strategy.

Mesenchymal stem cells (MSCs) have demonstrated considerable potential in regenerative medicine due to their immunomodulatory and tissue-reparative properties [10]. However, safety concerns, including tumorigenic risks and cell embolism, limit their clinical application [11]. Recent studies indicate that the therapeutic effects of MSCs are largely mediated by paracrine factors, particularly small extracellular vesicles (sEV) [12, 13]. MSC-derived sEV (MSC-sEV) inherit the therapeutic benefits of their parent cells while offering superior safety profiles, including reduced immunogenicity and tumorigenic potential [12–15]. Among various MSC sources, human umbilical cord-derived MSC-sEV (hucMSC-sEV) have emerged as a research focus due to their superior accessibility, lower immunogenicity, and robust immunomodulatory capabilities [16].

MSC-sEV have been reported to possess multiple biological functions, including inhibiting inflammatory responses [17–19], counteracting oxidative stress damage [20], and maintaining mitochondrial functional integrity [21]. Notably, studies reveal that the beneficial effects of MSC-sEV are predominantly attributed to their cargo of MSC-derived microRNAs (miRNAs) [22]. Furthermore, our previous research demonstrated the efficacy of sEV-based anti-inflammatory therapy targeting RTECs in ischemic AKI [23–25]. However, in the pathological context of S-AKI, the key effector molecules within the complex miRNA profile of MSC-sEV and their specific actions on RTECs remain unclear. Therefore, comprehensively evaluating the therapeutic role of MSC-sEV in S-AKI and elucidating the critical miRNAs mediating RTECs regulation are of paramount importance for future translational applications.

In this study, we demonstrated that MSC-sEV ameliorate S-AKI by delivering miR-125a-5p to RTECs, which specifically inhibits TNFR2, blocks TNF-activated NLRP3/GSDMD signaling pathways, and attenuates pyroptosis along with its driven inflammatory cascades. Furthermore, we developed an EXOmotif GGAG-based engineering strategy to enhance the selective loading of miR-125a-5p into sEV, thereby improving the therapeutic miRNA delivery efficiency of MSC-sEV. Overall, this study may pave the way for using MSC-sEV to treat the life-threatening S-AKI by targeting pyroptosis.

## Results

### MSC-sEV homes to injured kidneys

**Fig. 1:**
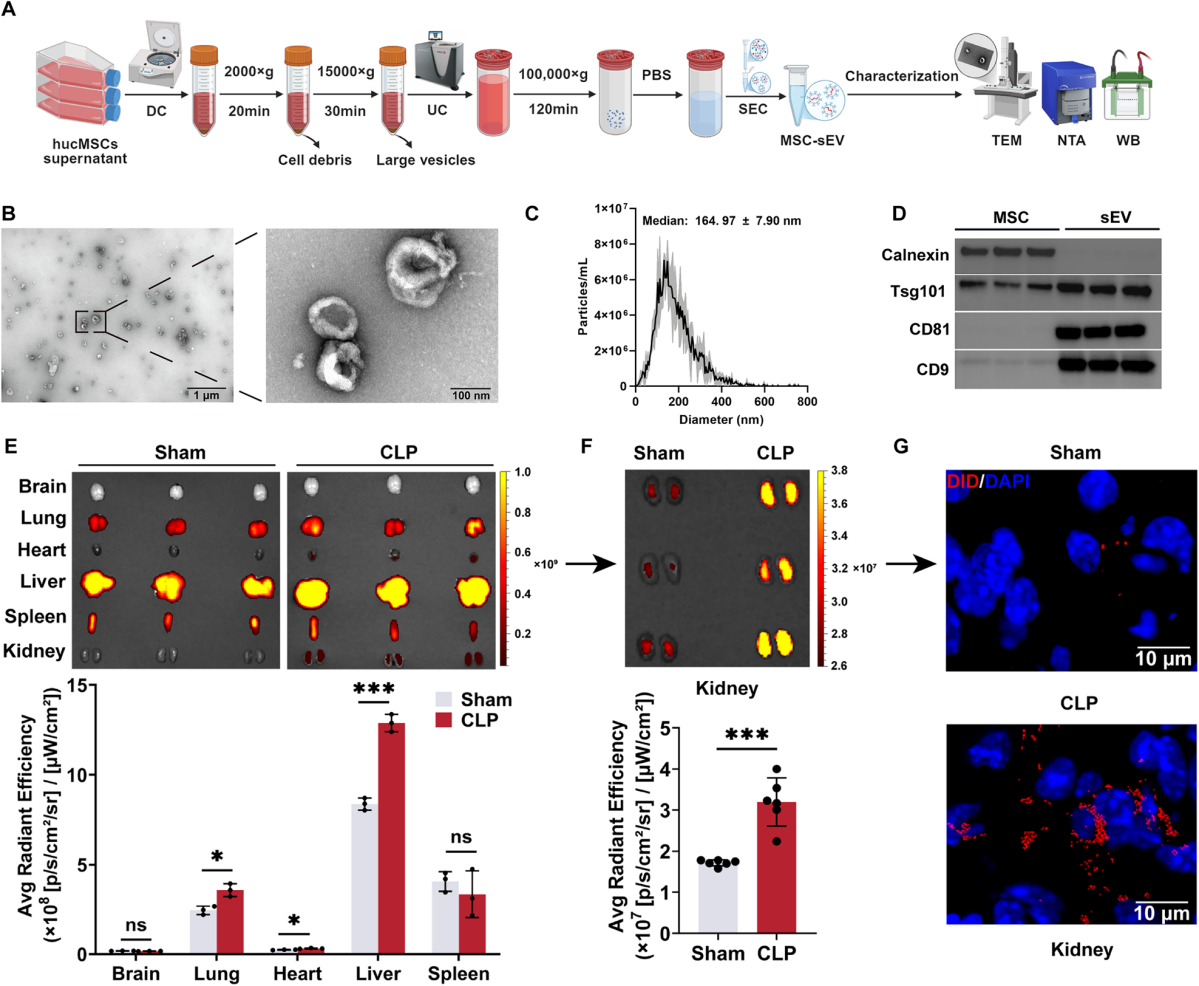
MSC-sEV homes to injured kidneys. **A** Isolation workflow combining dUC and SEC. **B** TEM images showing cup-shaped morphology of MSC-sEV. **C** NTA analysis of MSC-sEV size distribution. **D** Western blot detection of EVs markers. **E** IVIS imaging of DiD-MSC-sEV biodistribution in major organs. **F** Distribution of MSC-sEV in kidneys. **G** Frozen kidney sections showing tubular localization of DiD-MSC-sEV. Scale bar, 10 μm. Data are presented as mean ± SD. *n* = 3 per group. *P < 0.05, ***P < 0.001, ns, not significant (*P* > 0.05), unpaired, 2-tailed Student’s *t* test.

The isolated MSCs displayed spindle-shaped morphology with adherent growth and demonstrated osteogenic, adipogenic, and chondrogenic differentiation capacities (Fig. S1A–D). Flow cytometry confirmed surface marker expression: CD73, CD90, and CD105 positivity >95%, while HLA-DR, CD11b, CD19, CD34, and CD45 positivity <2% (Fig. S1E). MSC-sEV were isolated following the workflow in Fig. 1A. TEM revealed typical cup-shaped bilayer membrane vesicles (Fig. 1B). NTA indicated a median diameter of 164.97 ± 7.90 nm (Fig. 1C). Western blot confirmed expression of Tsg101, CD9, and CD81, with absence of Calnexin (Fig. 1D).

To investigate the biodistribution of MSC-sEV in sepsis, DiD-labeled MSC-sEV (DiD-MSC-sEV) were administered via tail vein. Ex vivo IVIS imaging showed significantly higher DiD fluorescence signals in lungs, heart, liver, and kidneys of CLP mice versus sham controls (Fig. 1E, F). Notably, CLP kidneys exhibited extensive DiD-MSC-sEV accumulation around RTECs (Fig. 1G). These results suggest that MSC-sEV can homing to the injured kidney.

### MSC-sEV protects the kidneys from sepsis-induced injury

**Fig. 2:**
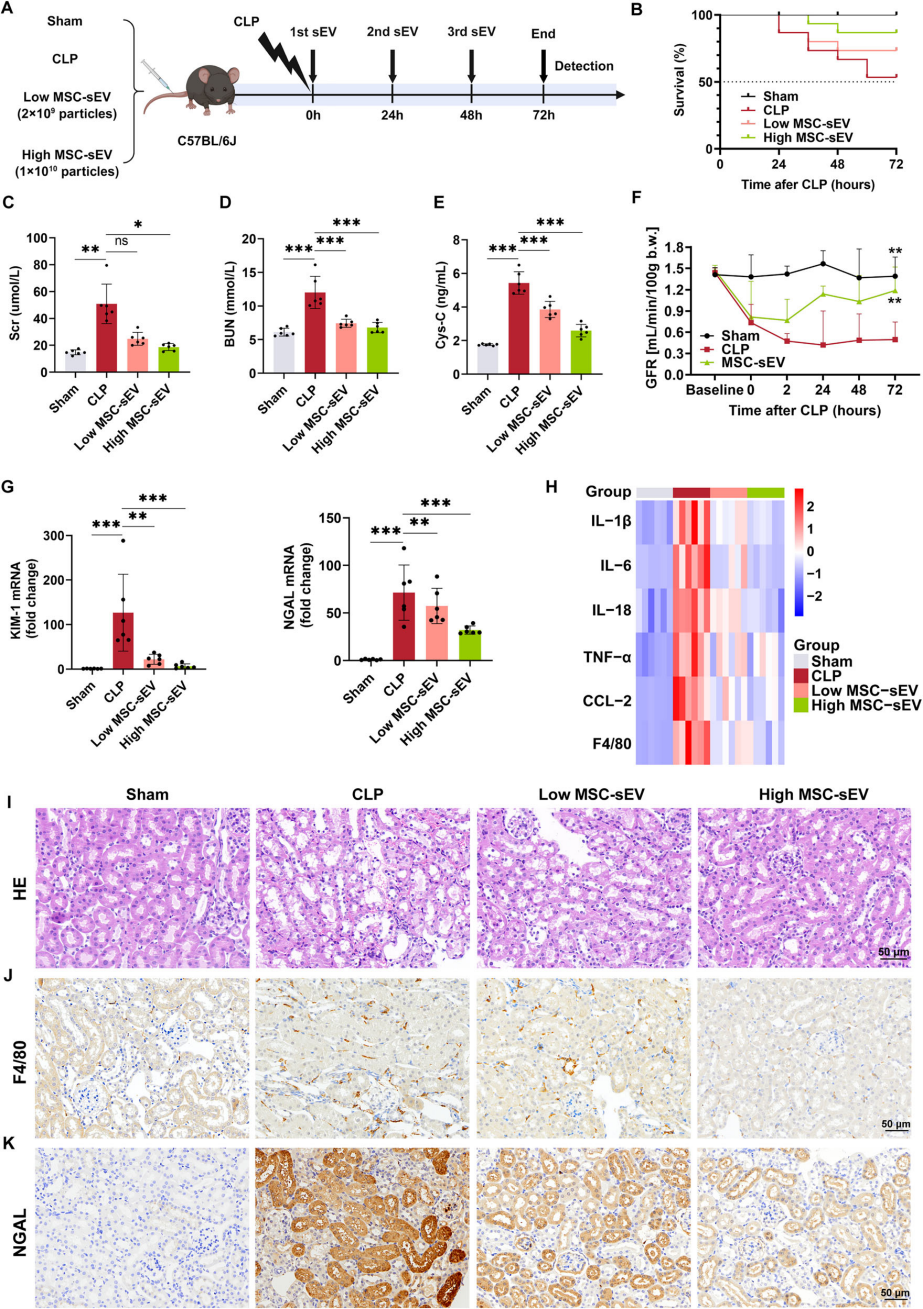
MSC-sEV protects the kidneys from sepsis-induced injury. **A** Animal experimental protocol. **B** Survival curves of mice (*n* = 15 per group). **C**–**E** Results of serum renal function indicators Scr, BUN and Cys-C. **F** Results of GFR changes over time (*n* = 3 per group). **G** KIM-1 and NGAL mRNA levels in renal tissues. **H** mRNA levels of pro-inflammatory cytokines and chemokines (IL-1β, IL-6, IL-18, TNF-α, CCL2, F4/80) in renal tissues. **I** Representative images of HE staining of renal tissue. Scale bar, 50 μm. **J**, **K** Representative images of F4/80 and NGAL immunohistochemical staining of renal tissue. Scale bar, 50 μm. Data are presented as mean ± SD. *P < 0.05, **P < 0.01, ***P < 0.001, ns, not significant (*P* > 0.05), one-way ANOVA.

The therapeutic effect of MSC-sEV on S-AKI was evaluated 72 h after CLP (Fig. 2A). Compared to the CLP group, MSC-sEV treatment dose-dependently improved survival rates, decreased serum creatinine (Scr), blood urea nitrogen (BUN), and cystatin C (Cys-C) levels, and enhanced glomerular filtration rate (GFR) in S-AKI mice (Fig. 2B–F). Moreover, qRT-PCR analysis revealed significantly lower mRNA levels of renal injury biomarkers (KIM-1 and NGAL) in MSC-sEV-treated groups, particularly the high-dose group, compared to CLP group (Fig. 2G). The same trend was observed in pro-inflammatory cytokines and chemokines (IL-1β, IL-6, IL-18, TNF-α, CCL2, and F4/80) (Fig. 2H).

Histopathological evaluation showed marked tubular epithelial cell detachment, cytoplasmic vacuolization, interstitial inflammatory cell infiltration, and vascular congestion in CLP group. Immunostaining confirmed elevated F4/80 and NGAL expression in renal tissues of the CLP group. Strikingly, MSC-sEV intervention substantially mitigated these pathological alterations (Fig. 2I–K). Collectively, these findings indicate that MSC-sEV attenuates renal inflammation, ameliorates kidney injury, and improves survival in S-AKI.

### MSC-sEV attenuates inflammatory and oxidative responses in LPS-stimulated HK-2 cells

**Fig. 3:**
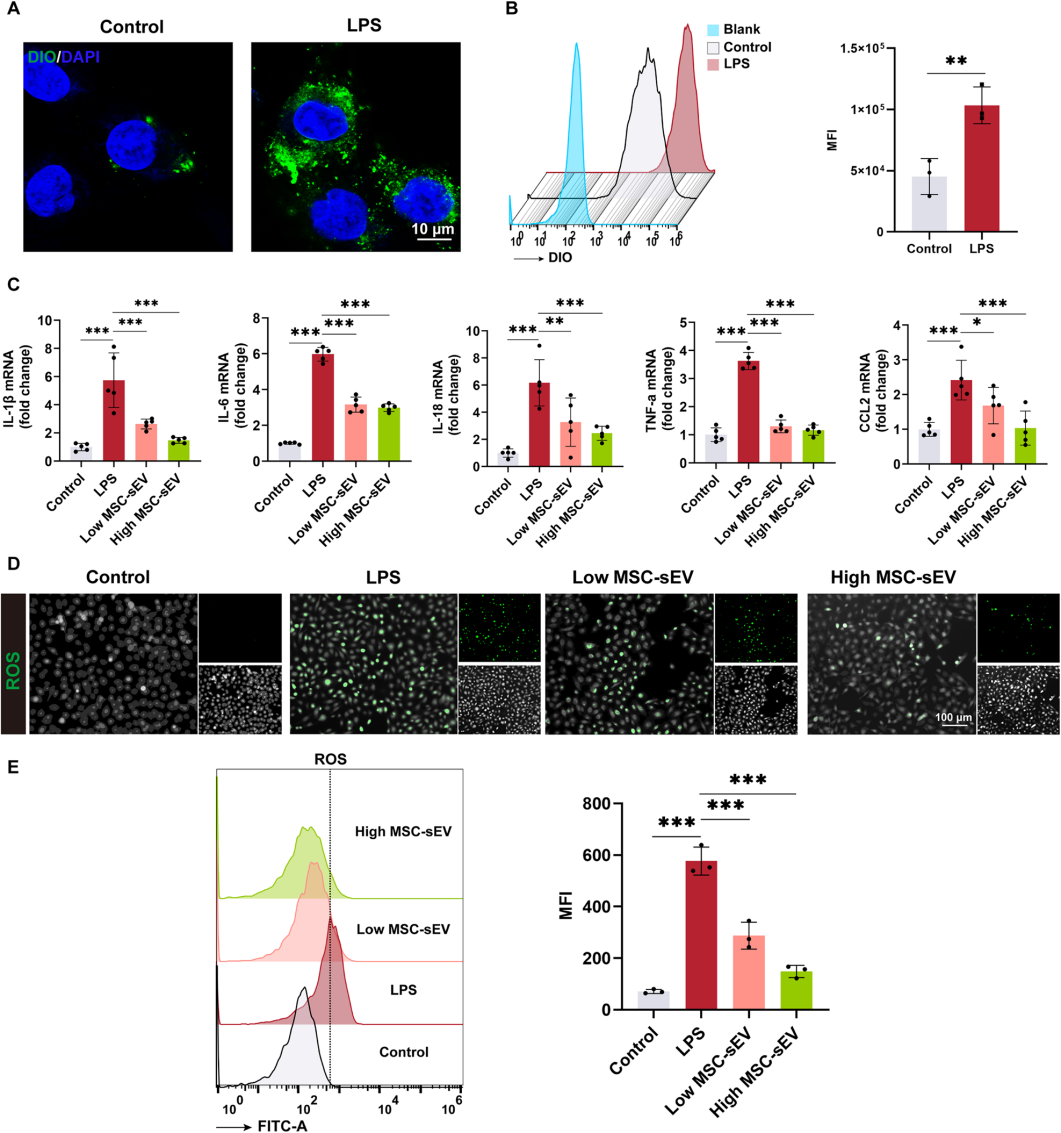
MSC-sEV attenuates inflammatory and oxidative responses in LPS-stimulated HK-2 cells. **A** Confocal microscopy to observe the uptake of MSC-sEV by HK-2 cells. Scale bar, 10 μm. **B** Flow cytometric quantification of MSC-sEV internalization in HK-2 cells. **C** mRNA levels of pro-inflammatory cytokines (IL-1β, IL-6, IL-18, TNF-α, CCL2) in HK-2 cells. **D** Representative fluorescence images of ROS in HK-2 cells. Scale bar, 100 μm. **E** Flow cytometry determination of ROS levels in HK-2 cells. Data are presented as mean ± SD. *P < 0.05, **P < 0.01, ***P < 0.001, unpaired, 2-tailed Student’s *t* test & one-way ANOVA.

We further validated MSC-sEV effects on RTECs using an LPS-induced in vitro S-AKI model. As shown in Fig. 3A, B, LPS-injured HK-2 cells exhibited enhanced DiO-MSC-sEV uptake. Flow cytometry confirmed significantly higher mean DiO fluorescence intensity in LPS-treated versus control cells, consistent with in vivo biodistribution patterns. Notably, MSC-sEV treatment dose-dependently attenuated LPS-induced inflammation and oxidative stress in HK-2 cells, as evidenced by reduced mRNA levels of IL-1β, IL-6, IL-18, TNF-α, CCL2, and decreased ROS production compared to LPS group (Fig. 3C–E). These results confirm that MSC-sEV contributes to ameliorate inflammation and oxidative damage in RTECs in vitro.

### MSC-sEV down-regulates TNFR2 to suppress NLRP3 inflammasome activation and attenuates inflammatory responses in S-AKI

**Fig. 4:**
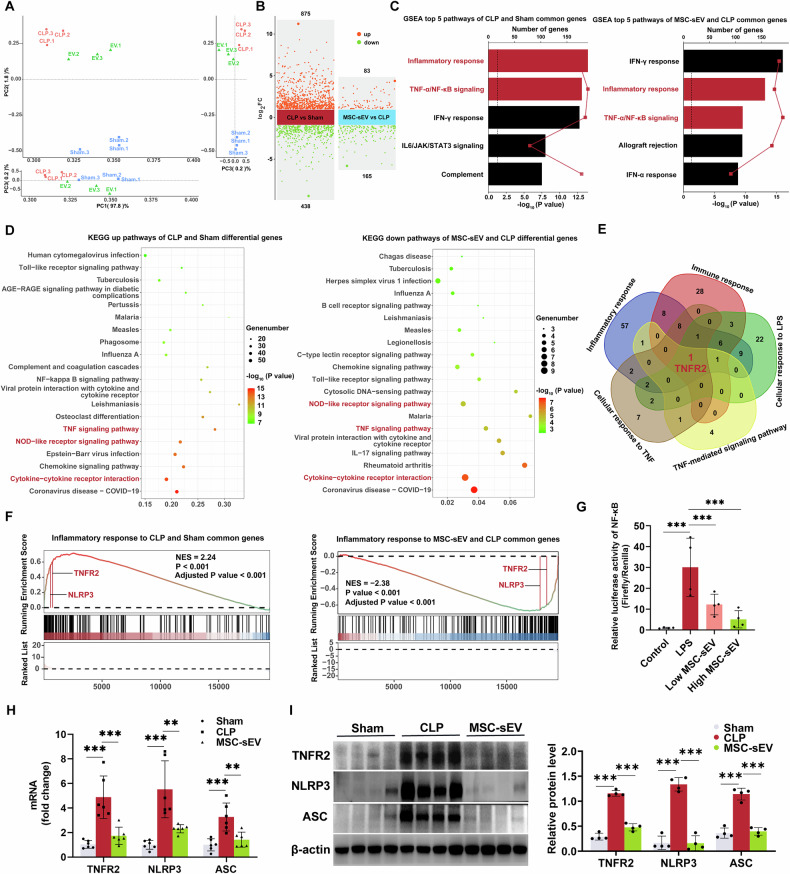
MSC-sEV down-regulates TNFR2 to suppress NLRP3 inflammasome activation and attenuates inflammatory responses in S-AKI. **A** Principal component analysis of renal transcriptomes. **B** Scatter plot of multiple sets of differential genes. **C** Top 5 pathways of GSEA analysis. **D** KEGG pathway enrichment analysis of differentially expressed genes. **E** Combined analysis of 5 key biological processes in sepsis. **F** Changes of TNFR2 and NLRP3 in inflammatory response pathway. **G** Relative luciferase activity of NF-κB. **H** mRNA levels of TNFR2, NLRP3, and ASC in renal tissues. **I** Western blot quantification of TNFR2, NLRP3, and ASC proteins expression (*n* = 4 per group). Data are presented as mean ± SD. **P < 0.01, ***P < 0.001, one-way ANOVA.

To elucidate the molecular mechanisms underlying MSC-sEV-mediated renal protection in S-AKI, mRNA sequencing of renal tissues was performed followed by bioinformatic analyses. Principal component analysis and volcano plots revealed distinct clustering and significant differential gene expression between groups (Fig. 4A, B). The sequencing results of mRNA were verified by qRT-PCR and showed a significant positive correlation (Fig. S2A). Notably, Gene Set Enrichment Analysis (GSEA) identified inflammation-related pathways as the most significantly up-regulated in S-AKI, with TNF-α/NF-κB signaling ranking second. Both pathways were markedly down-regulated by MSC-sEV intervention (Fig. 4C). Consistently, KEGG pathway analysis demonstrated that cytokine-cytokine receptor interactions, TNF signaling, and NOD-like receptor signaling were predominantly up-regulated post-CLP and suppressed by MSC-sEV treatment (Fig. 4D). In particular, to identify core regulatory genes in S-AKI pathogenesis, we integrated five biological processes central to sepsis pathophysiology: inflammatory response, immune response, cellular response to LPS, TNF response, and TNF-mediated signaling. TNFR2 (a membrane-bound TNF receptor) emerged as the sole hub gene co-regulating all five processes, showing CLP-induced upregulation and MSC-sEV-driven down-regulation (Fig. 4E, Fig. S2B-C).

Further, among the changes in the inflammatory response, we found that TNFR2 and NLRP3—key mediators of TNF and NOD-like receptor pathways, respectively—were up-regulated in CLP mice but down-regulated by MSC-sEV (Fig. 4F). Critically, to validate MSC-sEV-mediated suppression of inflammatory responses, 293 T cells transfected with hNF-κB promoter-Luc were stimulated with LPS and treated with MSC-sEV. As demonstrated in Fig. 4G, LPS stimulation markedly increased NF-κB luciferase activity, whereas MSC-sEV intervention dose-dependently attenuated this activation. Then, to verify the reliability of the transcriptomics results, we performed qRT-PCR and Western blot experiments. The results indicate that both mRNA and protein levels of TNFR2, NLRP3, and ASC were elevated post-CLP but significantly reduced by MSC-sEV intervention (Fig. 4H–I). Previous studies have established TNFR2 promotes NLRP3 inflammasome activation via the NF-κB pathway [26–28]. These findings collectively demonstrate that MSC-sEV attenuates renal inflammation by suppressing NLRP3 inflammasome activation through TNFR2 down-regulation.

### MSC-sEV alleviates renal inflammatory injury by suppressing RTECs pyroptosis in S-AKI

**Fig. 5:**
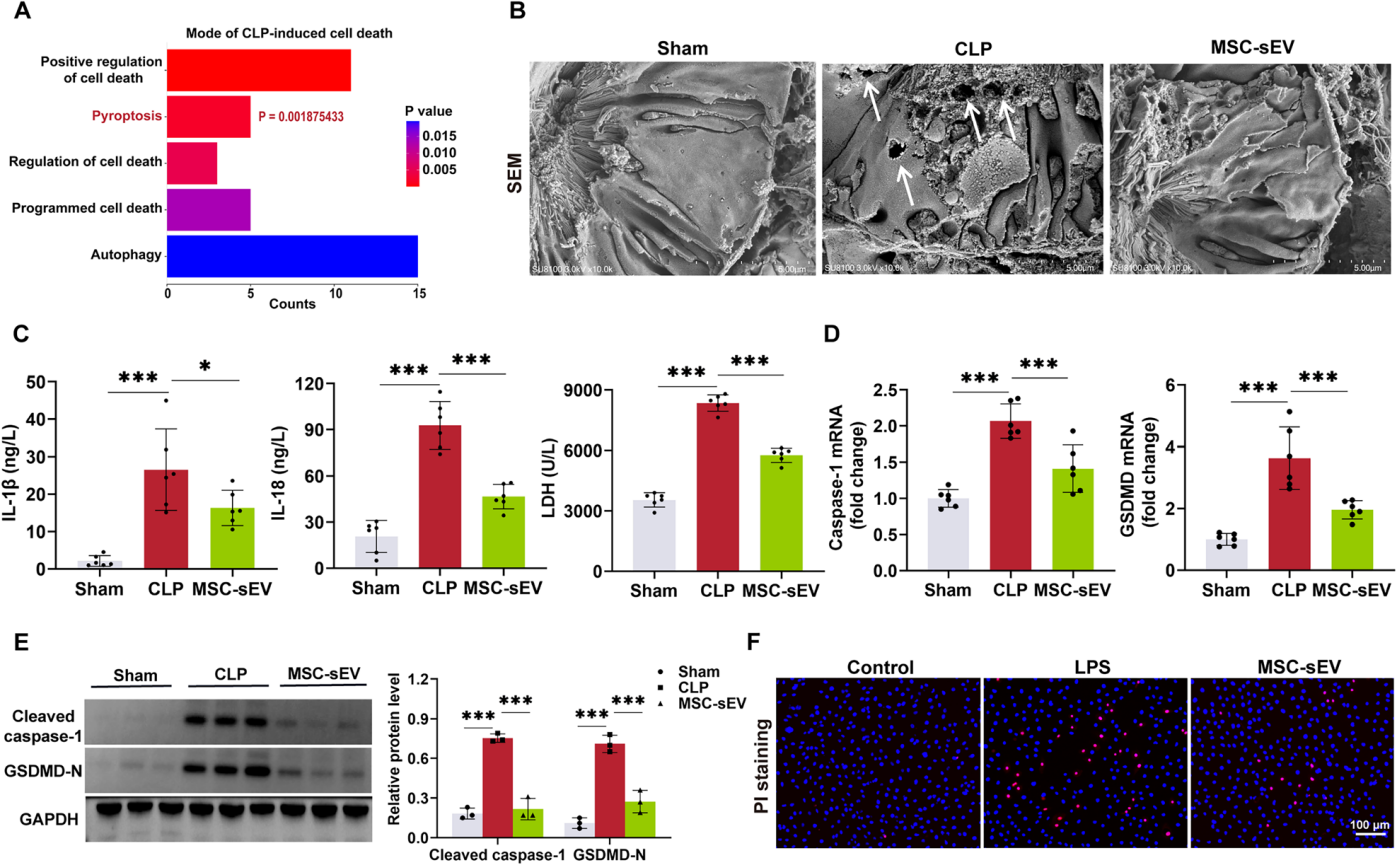
MSC-sEV alleviates renal inflammatory injury by suppressing RTECs pyroptosis in S-AKI. **A** GO annotation analysis revealed the central role of pyroptosis in S-AKI. **B** Representative morphological images of RTECs under scanning electron microscopy. White arrows indicate membrane pores. Scale bar, 5 μm. **C** Serum levels of IL-1β, IL-18, and LDH. **D** mRNA levels of Caspase-1 and GSDMD in renal tissues. **E** Western blot quantification of Cleaved caspase-1 and GSDMD-N proteins expression (*n* = 3 per group). **F** Representative images of propidiumIodide staining of HK-2 cells. Scale bar, 100 μm. Data are presented as mean ± SD. *P < 0.05, ***P < 0.001, one-way ANOVA.

In the kidney, RTECs death is a pivotal driver of AKI pathogenesis [29]. Building on our findings, we analyzed transcriptomic data to explore the association between MSC-sEV-induced TNFR2 down-regulation and RTECs phenotypic regulation. Pyroptosis emerged as the most significantly enriched programmed cell death pathway (Fig. 5A). Notably, pyroptosis—an inflammatory cell death—triggers inflammatory cascades and exacerbates tissue injury. In the study, SEM imaging revealed characteristic plasma membrane pore formation indicative of pyroptosis in RTECs in the CLP group, whereas MSC-sEV-treated cells maintain membrane integrity (Fig. 5B). Furthermore, serum levels of IL-1β, IL-18, and LDH were markedly elevated in CLP group but significantly reduced by MSC-sEV intervention (Fig. 5C). Mechanistically, kidneys in the CLP group showed up-regulated caspase-1 and GSDMD mRNA expression, accompanied by increased cleaved caspase-1 and GSDMD-N levels at the protein level, all of which were suppressed by MSC-sEV (Fig. 5D-E). PI staining showed that PI^+^HK-2 cells were significantly increased in the LPS group and a decrease in the MSC-sEV group (Fig. 5F). Thus, these results collectively indicate that MSC-sEV mitigates RTECs injury primarily through pyroptosis suppression.

### MSC-sEV protects RTECs from pyroptosis by delivering miR-125a-5p to down-regulate TNFR2

**Fig. 6:**
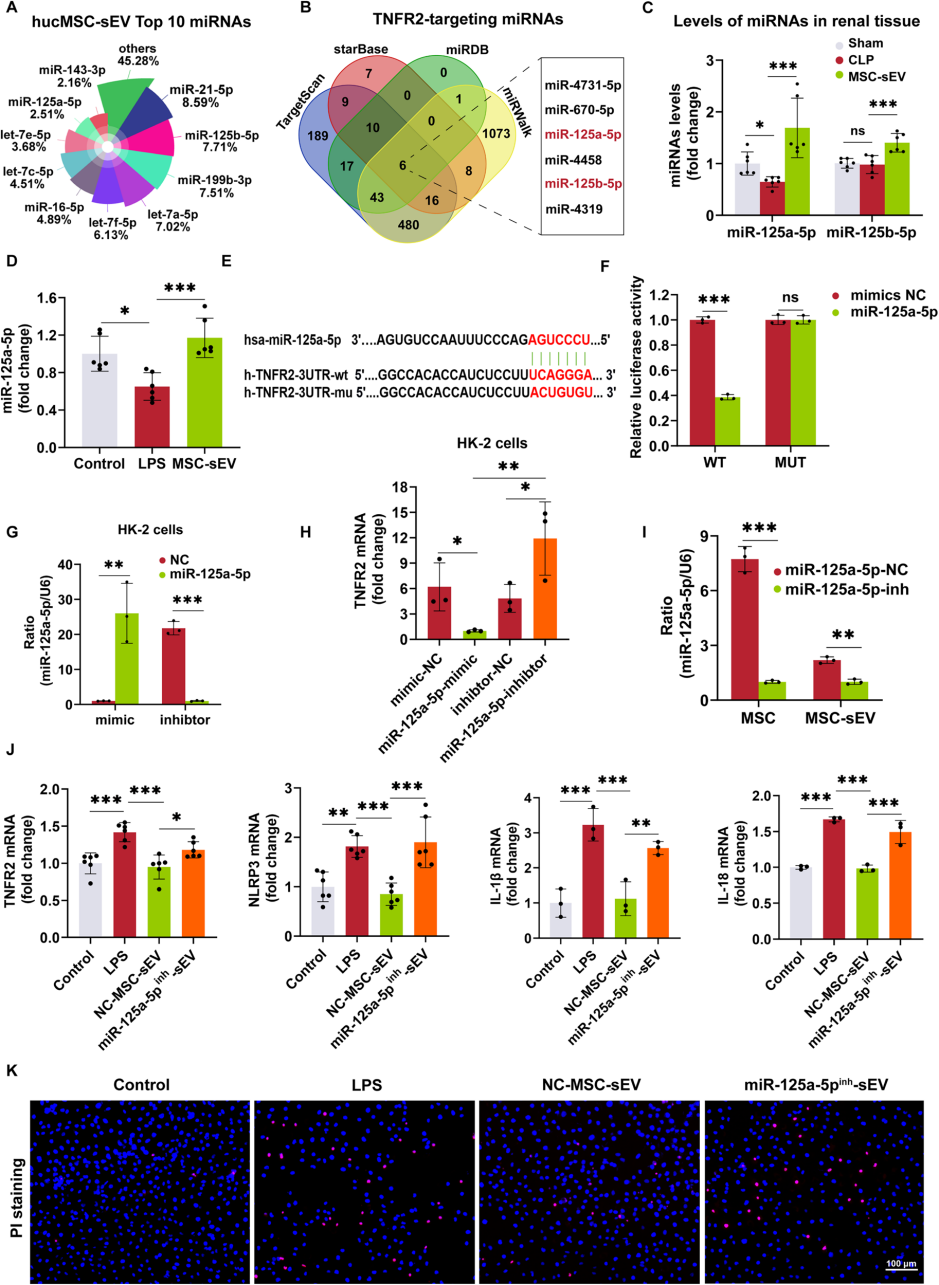
MSC-sEV protects RTECs from pyroptosis by delivering miR-125a-5p to down-regulate TNFR2. **A** Top 10 miRNAs in miRNAs sequencing profiles (*n* = 4). **B** Bioinformatics prediction of TNFR2-targeting miRNAs. **C** qRT-PCR detection of miRNAs levels in renal tissues. **D** qRT-PCR detection of miRNA levels in HK-2 cells. **E** Binding sites of miR-125a-5p to TNFR2. **F** Dual-luciferase reporter assay validating miR-125a-5p targeting TNFR2. **G**, **H** miR-125a-5p and TNFR2 expression in HK-2 cells transfected with miR-125a-5p mimic and inhibitor. **I** Levels of miR-125a-5p in MSCs and MSC-sEV after miR-125a-5p-inhibtor transfection of MSCs. **J** TNFR2, NLRP3, IL-1β and IL-18 expression in HK-2 cells treated with miR-125a-5p-inhibited MSC-sEV. **K** Representative images of propidiumIodide staining of HK-2 cells. Scale bar, 100 μm. Data are presented as mean ± SD. *P < 0.05, **P < 0.01, ***P < 0.001, ns, not significant (*P* > 0.05), one-way ANOVA.

Our findings identified TNFR2 as a key S-AKI gene suppressed by MSC-sEV. To pinpoint critical miRNAs mediating this effect, miRNA sequencing of MSC-sEV revealed 813 co-expressed miRNAs across four independent samples. The top 10 most abundant miRNAs—miR-21-5p, miR-125b-5p, miR-199b-3p, let-7a-5p, let-7f-5p, miR-16-5p, let-7c-5p, let-7e-5p, miR-125a-5p, and miR-143-3p—collectively accounted for 54.72% of total miRNA content (Fig. 6A). Integrated analysis using TargetScan, starBase, miRDB, and miRWalk databases identified eight miRNAs targeting TNFR2, including two from the top 10 list: miR-125a-5p and miR-125b-5p (Fig. 6B). qRT-PCR validation revealed significant upregulation of both miR-125a-5p and miR-125b-5p in MSC-sEV-treated kidneys. Notably, only miR-125a-5p was differentially down-regulated in the CLP groups. In vitro, miR-125a-5p exhibited a trend consistent with renal tissues (Fig. 6C-D).

Bioinformatic analysis predicted multiple binding sites between miR-125a-5p and TNFR2, which was experimentally validated by dual-luciferase reporter assays confirming that miR-125a-5p targets and downregulates TNFR2 (Fig. 6E, F). Transfection of miR-125a-5p mimics into HK-2 cells down-regulated TNFR2, while miR-125a-5p inhibitors up-regulated it (Fig. 6G, H). Similarly, MSCs were transfected by miR-125a-5p inhibtor and sEV (miR-125a-5p^inh^-sEV) was collected for interfering HK-2 cells. As shown in Fig. 6I–J, markedly reduced miR-125a-5p levels were observed in both MSCs and miR-125a-5p^inh^-sEV, whereas TNFR2, NLRP3, IL-1β, and IL-18 levels were significantly elevated in miR-125a-5p^inh^-sEV-treated HK-2 cells compared to NC-MSC-sEV group. In addition, the PI-positive rate of HK-2 cells in the miR-125a-5p^inh^-sEV group was also significantly elevated (Fig. 6K). Thus, these results conclusively demonstrate MSC-sEV protects RTECs from pyroptosis by targeting downregulation of TNFR2 through delivery of miR-125a-5p.

### Inhibition of miR-125a-5p diminishes the ability of MSC-sEV to downregulate TNFR2, thereby exacerbating pyroptosis in RTECs

**Fig. 7:**
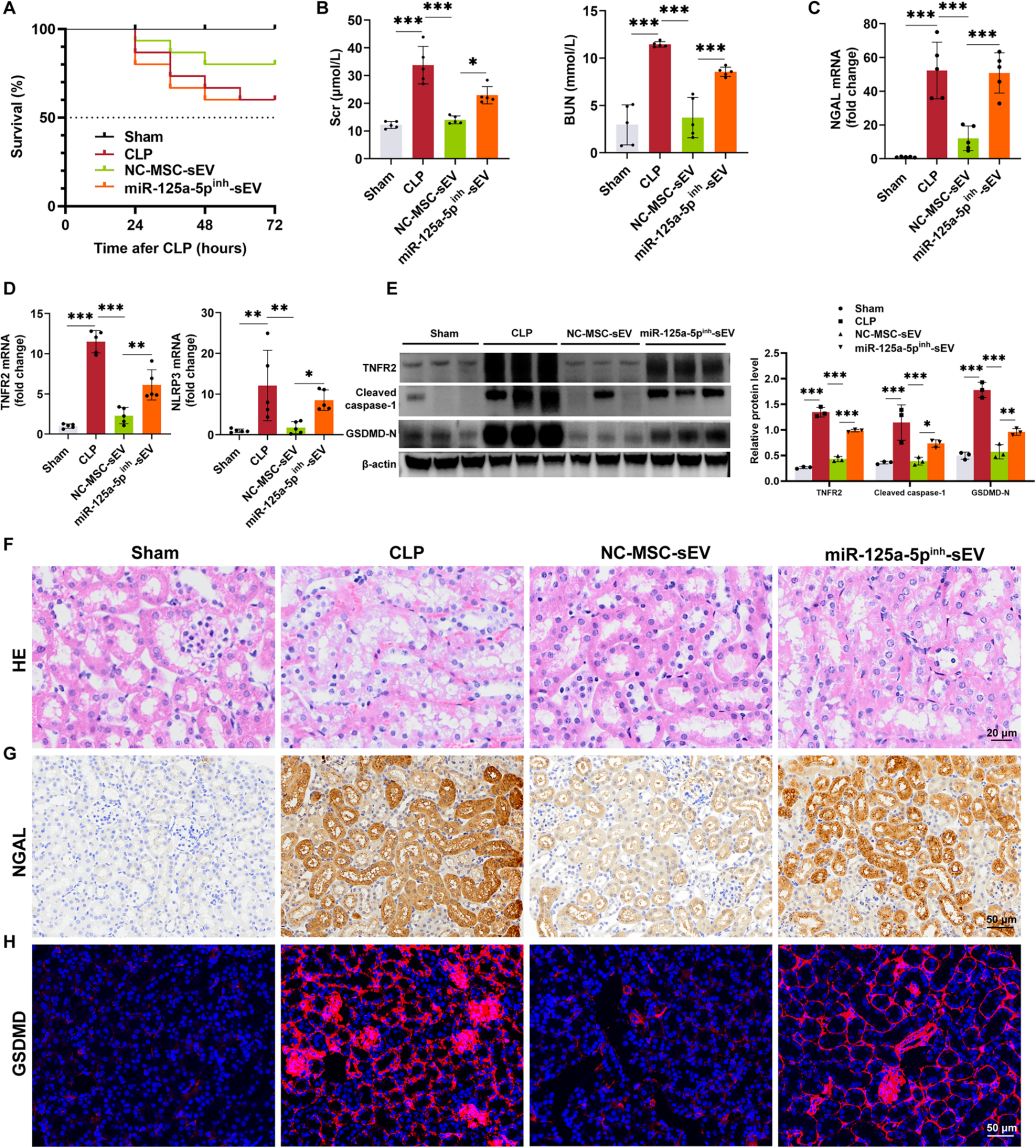
Inhibition of miR-125a-5p attenuates MSC-sEV down-regulation of TNFR2 exacerbating RTECs pyroptosis. **A** Survival curves of mice (*n* = 15 per group). **B** Results of serum Scr and BUN. **C**, **D** NGAL, TNFR2 and NLRP3 mRNA levels in renal tissues. **E** Western blot quantification of TNFR2, Cleaved caspase-1 and GSDMD-N proteins expression (*n* = 3 per group). **F** Representative images of HE staining of renal tissues. Scale bar, 20 μm. **G** Representative images of NGAL immunohistochemical staining of renal tissue. Scale bar, 50 μm. **H** Representative images of renal tissue immunofluorescence staining of GSDMD. Scale bar, 50 μm. Data are presented as mean ± SD. *P < 0.05, **P < 0.01, ***P < 0.001, one-way ANOVA.

To further confirm the renoprotective effect of MSC-sEV on RTECs pyroptosis blockade via miR-125a-5p, we compared the efficacy of NC-MSC-sEV with miR-125a-5p^inh^-sEV in vivo. Compared to the NC-MSC-sEV group, miR-125a-5p^inh^-sEV treatment resulted in reduced survival rates, elevated Scr and BUN levels, increased renal NGAL mRNA expression (Fig. 7A–C). Furthermore, TNFR2, NLRP3, Cleaved caspase-1 and GSDMD-N levels were significantly up-regulated in miR-125a-5p^inh^-sEV-treated kidneys compared to NC-MSC-sEV group (Fig. 7D, E). Histopathologically, tubular damage was evident in the miR-125a-5p^inh^-sEV group, with increased protein expression of both NGAL and GSDMD (Fig. 7F–H). The results demonstrate that inhibition of miR-125a-5p reverses MSC-sEV-mediated TNFR2 suppression, leading to RTECs pyroptosis and increased kidney injury.

### EXOmotif GGAG modification enhances miR-125a-5p loading in MSC-sEV and bolsters RTECs resistance to pyroptosis

**Fig. 8:**
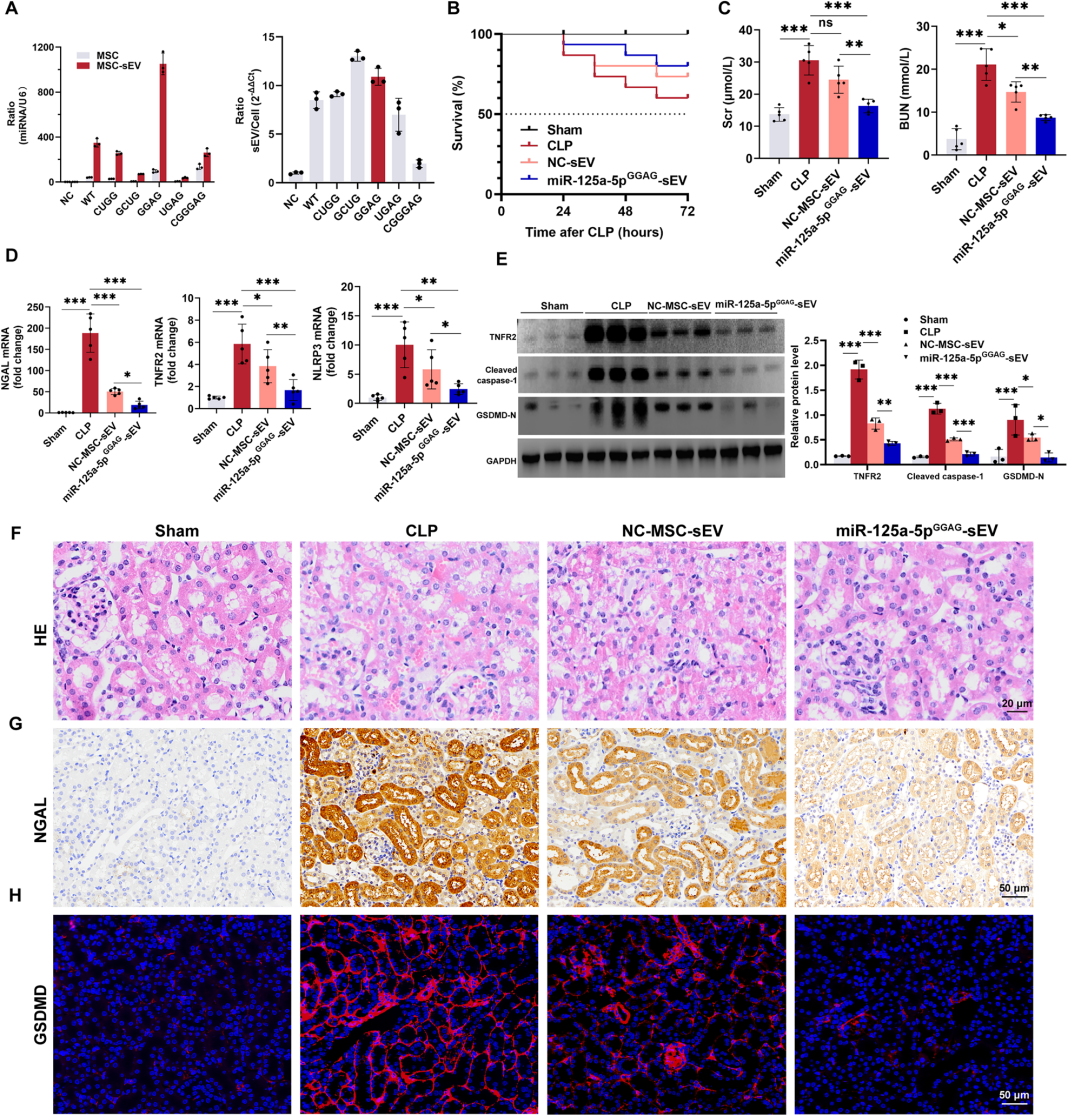
EXOmotif GGAG modification enhances miR-125a-5p loading in MSC-sEV and bolsters RTECs resistance to pyroptosis. **A** Effects of five different EXOmotifs on miRNA loading efficiency in MSC-sEV (*n* = 3 per group). **B** Survival curves of mice (*n* = 15 per group). **C** Results of serum Scr and BUN. **D** NGAL, TNFR2 and NLRP3 mRNA levels in renal tissues. **E** Western blot quantification of TNFR2, Cleaved caspase-1 and GSDMD-N proteins expression (*n* = 3 per group). **F** Representative images of HE staining of renal tissues. Scale bar, 20 μm. **G** Representative images of NGAL immunohistochemical staining of renal tissue. Scale bar, 50 μm. **H** Representative images of renal tissue immunofluorescence staining of GSDMD. Scale bar, 50 μm. Data are presented as mean ± SD. *P < 0.05, **P < 0.01, ***P < 0.001, ns, not significant (*P* > 0.05), one-way ANOVA.

Our findings identified miR-125a-5p as the key bioactive component mediating MSC-sEV renoprotection in S-AKI. To optimize therapeutic efficacy, miR-125a-5p was engineered with EXOmotifs—short nucleotide sequences enhancing miRNA loading efficiency. Based on the previous studies [30–33], five candidated EXOmotifs (CUGG, GCUG, GGAG, UGAG, CGGGAG) were transfected into MSCs, with qRT-PCR confirming GGAG as the most potent enhancer of miRNA sorting into sEV (Fig. 8A). As shown in Fig. S3, we found that GGAG modification significantly increased the miR-125a-5p content in sEV by more than 20-fold compared to WT modification, and by more than 30-fold compared to naturally produced MSC-sEV. GGAG-modified miR-125a-5p-loaded MSC-sEV (miR-125a-5p^GGAG^-sEV) were then evaluated in vivo. At equivalent doses, miR-125a-5p^GGAG^-sEV outperformed NC-MSC-sEV, significantly improving survival rates and reducing Scr and BUN levels in septic mice (Fig. 8B, C). Furthermore, miR-125a-5p^GGAG^-sEV demonstrated superior suppression of NGAL, TNFR2, NLRP3, Cleaved caspase-1 and GSDMD-N levels (Fig. 8D, E). Consistently, miR-125a-5p^GGAG^-sEV more significantly attenuated renal histopathological injury and reduced NGAL and GSDMD protein expression (Fig. 8F–H). These results demonstrate that GGAG modification significantly improves miR-125a-5p loading efficiency in MSC-sEV, enhances the inhibition of RTECs pyroptosis, and thereby more effectively rescues S-AKI.

## Discussion

**Fig. 9:**
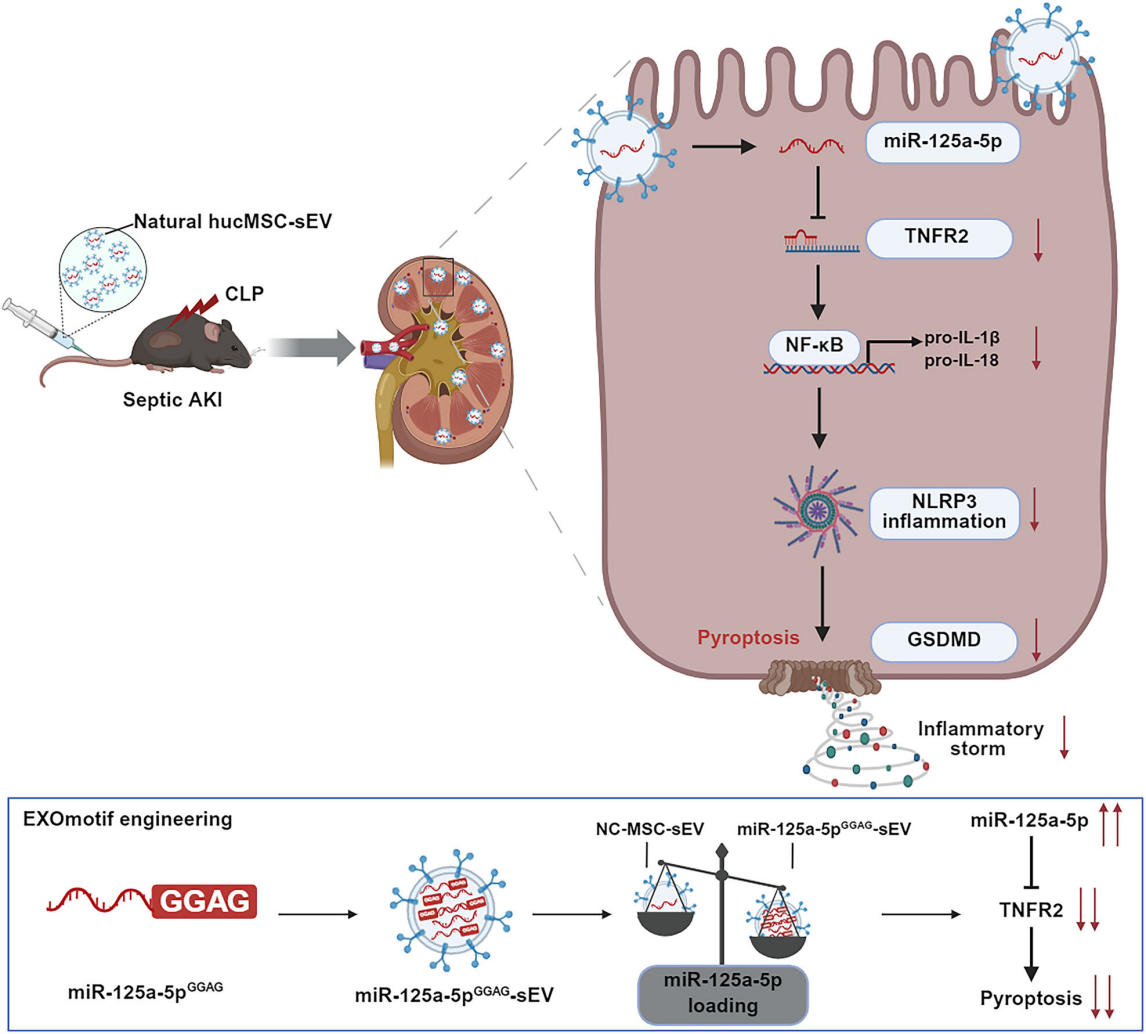
Schematic representation of the mechanism of miR-125a-5p enriched MSC-sEV against S-AKI. MSC-sEV can deliver miR-125a-5p into RTECs and suppress TNFR2 expression. Reduced TNFR2 decreases activation of the NF-κB/NLRP3/GSDMD axis, reducing pyroptosis.

AKI is a serious complication of sepsis with high morbidity and mortality [34]. Current clinical management remains constrained by the absence of targeted therapeutics. Infection-induced cytokine storm is widely recognized as a pivotal driver of multi-organ dysfunction in S-AKI pathogenesis [35]. Consequently, innovative strategies modulating inflammatory responses hold significant therapeutic potential. In this study, we demonstrated that MSC-sEV significantly suppress pro-inflammatory cytokine production, improve renal function, and enhance survival in S-AKI. Importantly, we elucidated a novel mechanism wherein MSC-sEV-derived miR-125a-5p targets TNFR2 to inhibit NLRP3 inflammasome activation, thereby attenuating RTECs pyroptosis and disrupting inflammatory cascades. Intriguingly, EXOmotif GGAG engineering was shown to markedly enhance miR-125a-5p loading efficiency in MSC-sEV, amplifying their renoprotective efficacy (Fig. 9). These findings may pioneer new therapeutic paradigms for S-AKI management.

Cell death is increasingly recognized as a key driver of inflammatory diseases, with cell death blockade demonstrating potential to reverse inflammatory pathologies in both acute and chronic disorders [36]. Pyroptosis, a pro-inflammatory cell death modality ubiquitously present across cell types [37], triggers inflammatory cascades via pore-driven cytoplasmic content release, exacerbating cytokine storms and multi-organ damage [38]. Although S-AKI pathophysiology remains incompletely understood, emerging evidence implicates pyroptosis-driven RTECs injury in disease progression [39–41]. TNF, a master regulator of inflammatory responses, not only directly activates pro-inflammatory gene expression but also indirectly amplifies inflammation by inducing lytic cell death [42]. Studies confirm TNF promotes pyroptosis via inflammasome activation, critically sustaining inflammatory responses [43–46]. However, the regulatory interplay between TNF-mediated pyroptosis and MSC-sEV intervention in S-AKI remains unexplored. Our study demonstrates that TNF activates the NLRP3/GSDMD axis via TNFR2 to induce RTEC pyroptosis and aggravate renal injury in S-AKI, while MSC-sEV mitigate these effects through TNFR2-specific suppression. Notably, while the clinical efficacy of anti-TNF biologics is attributed to blocking TNF binding to its cognate receptors (TNFR1/TNFR2), therapeutic mechanisms have historically focused on TNFR1 inhibition, with TNFR2’s role remaining understudied. This work identifies TNFR2 as a novel therapeutic target for TNF-associated pyroptosis, delineating the TNF-TNFR2 axis as a central mediator of S-AKI pathogenesis. These findings expand understanding of MSC-EVs-mediated renoprotection, particularly its capacity to inhibit TNF-driven pyroptotic pathways, from a receptor selectivity perspective.

MSC-sEV are polyfunctional molecular complexes secreted by MSCs, regulating biological processes through targeted delivery of bioactive cargo [47]. However, their key functional components vary across disease contexts. Our previous studies have confirmed that the membrane proteins VLA-4 and LFA-1 on MSC-sEV mediate their homing property through interactions with VCAM-1 and ICAM-1 expressed on injured RTECs, thereby enabling targeted delivery [19]. Interestingly, MSC-sEV have been found to contain abundant miRNAs with high regulatory activity [22, 25]. Here, miRNA sequencing and experimental validation identified miR-125a-5p, a top 10 abundant miRNA in MSC-sEV—as being significantly reduced in both in vivo and in vitro S-AKI models, yet restored upon MSC-sEV intervention. Crucially, miR-125a-5p directly mediates targeted down-regulation of TNFR2. These findings suggest that miR-125a-5p may be the principal bioactive agent underlying MSC-sEV renoprotection in S-AKI. Further experiments demonstrated that miR-125a-5p-mediated TNFR2 suppression potently inhibits TNF-driven NLRP3/GSDMD signaling, thereby alleviating RTECs pyroptosis and improving renal dysfunction. Consistent with previous studies, miR-125a-5p has regulatory cell death and anti-inflammatory effects [48–50]. In contrast, this study uniquely focuses on S-AKI’s distinct pathological microenvironment, revealing that miR-125a-5p-enriched MSC-sEV rescue renal injury by regulating the pyroptotic phenotype of RTECs. This work expands the functional understanding of miR-125a-5p in S-AKI and provides mechanistic validation for sEV-based miRNA delivery strategies.

miRNAs are potent regulatory molecules capable of modulating entire cellular pathways through interactions with multiple target genes [51]. miRNA-based sEV therapy represents an advanced strategy that not only enables efficient delivery of therapeutic miRNAs to target cells with minimal off-target effects but also reduces immunogenic risks [52]. However, the low endogenous abundance of specific miRNAs within sEV poses a major translational barrier, as most individual vesicles lack biologically meaningful miRNA quantities [53], often necessitating high therapeutic doses [54]. To address this critical limitation, we employed EXOmotif engineering. GGAG modification was confirmed to robustly enhance miR-125a-5p loading into MSC-sEV. Significantly, at equivalent doses, miR-125a-5p-GGAG-sEV demonstrated superior renoprotection compared to native MSC-sEV, correlating with amplified TNFR2 down-regulation and potent suppression of RTECs pyroptosis. These findings validate that EXOmotif engineering optimizes therapeutic efficacy while reducing dosage requirements, thereby mitigating potential dose-dependent toxicity. Our approach of enhancing miRNA loading via EXOmotifs provides an innovative solution to overcome key challenges in sEV-based therapeutics.

Cell death-driven inflammation serves as a critical antimicrobial mechanism [36], while TNF, a central inflammatory cytokine, drives or exacerbates inflammatory injury by mediating cell death. Thus, cell death inhibitors have emerged as novel therapeutics for TNF-dependent inflammatory disorders [42]. This study demonstrates that TNF induces RTEC pyroptosis via the TNFR2/NLRP3 axis in S-AKI, whereas MSC-sEV block this process through miR-125a-5p delivery.

This study has several limitations. First, we focused primarily on a single signaling axis and one mode of cell death. However, given the complexity of biological systems, the potential involvement of other cell death modalities and signaling pathways cannot be completely excluded. Second, the bioactive components of MSC-sEV are highly diverse and may act synergistically, whereas this study investigated only one specific miRNA, which may not fully represent the overall biological effects of MSC-sEV. Third, the lack of positive pharmacological controls and clinical validation limits the translational generalizability of our findings. Future studies integrating multi-pathway analyses, multi-component validation, and clinical investigations are warranted to further substantiate these conclusions.

## Conclusions

We demonstrate that TNFR2 drives RTECs pyroptosis and inflammation in S-AKI. MSC-sEV deliver miR-125a-5p to suppress TNFR2, thereby inhibiting NLRP3 inflammasome, alleviating pyroptosis, and improving renal function. Interestingly, EXOmotif GGAG engineering enhanced miR-125a-5p loading in MSC-sEV, which potentiated their efficacy. These findings highlight MSC-sEV as a novel therapeutic strategy for S-AKI.

## Materials and methods

### Isolation, cultivation, and characterization of MSCs

As previously described [55], MSCs were isolated using the tissue explant method and maintained in static culture with MSC-specific medium (Sartorius) at 37 °C with 5% CO₂ [56]. Cells were passaged when reaching 80% confluence. For cellular identification, third-passage MSCs were subjected to flow cytometry (BD FACS Calibur™, USA) to detect surface markers including HLA-DR, CD11b, CD19, CD34, CD45, CD73, CD90, and CD105. Additionally, third-passage MSCs were induced for adipogenic, osteogenic, and chondrogenic differentiation following manufacturer protocols. After 3 weeks, Oil Red O, Alizarin Red, and Alcian Blue staining were performed respectively, with microscopic observation and imaging conducted using an Olympus microscope (Japan).

### Isolation and characterization of sEV

When MSCs reached the sixth passage at 80% confluence, the MSC-specific medium was replaced with exosome-depleted medium and cultured for an additional 48 h. Subsequently, cell supernatants were collected for sEV isolation via differential ultracentrifugation. Briefly, supernatants were centrifuged at 2000 × *g* for 20 min, 15000 × *g* for 30 min, and 100,000 × *g* for 120 min (Beckman, USA). The pellets were resuspended in pre-cooled sterile PBS. Following the manufacturer’s instructions, the suspension was filtered through a size-exclusion column (Enzeekon Technologies Co., China), and specific fractions containing purified MSC-sEV were collected, aliquoted, and stored at –80 °C.

To characterize the obtained sEV, the morphology was observed by transmission electron microscopy (TEM; HITACHI), particle size and Zeta potential were detected by nanoparticle tracking analysis (NTA; ZetaView PMX 110), surface molecules CD9, CD63 and CD81 of sEV were detected by nano-flow cytometry, and by Western blot detection of markers, including sEV positive marker proteins (Tsg101, CD9, CD81) and negative marker protein Calnexin.

### Establishment of S-AKI mouse model and MSC-sEV intervention

Specific pathogen free male C57BL/6 mice aged 6- to 8-week-old were purchased from Changzhou Cavens Laboratory Animal Co., Ltd. Mice were housed under standard conditions with free access to food and water and were randomly assigned to groups using a random number table. All animal procedures were performed in accordance with the Guide for the Care and Use of Laboratory Animals and approved by the Animal Care Committee of Southeast University (No. 20220224039). The work has been reported in line with the ARRIVE guidelines 2.0. Sepsis was induced via cecal ligation and puncture (CLP) as previously described [57]. Due to its ability to closely mimic the pathophysiological features of clinical sepsis, the CLP model is currently recognized internationally as the gold standard for sepsis animal models. Sham-operated mice underwent identical procedures without cecal ligation or puncture. Previous studies indicated that mortality in this model began within 18–24 h post-CLP, with the peak of death occurring within the first 72 h [57]. Therefore, we set 72 h post-CLP as the experimental endpoint. At 0, 24, and 48 h post-CLP, mice received tail vein injections of PBS (equal volume), low-dose MSC-sEV (2 × 10⁹ particles), or high-dose MSC-sEV (1 × 10¹⁰ particles). Fifteen mice per group were monitored regularly for survival rates and incidence of S-AKI. All animals were kept in the same environment. At 72 h after CLP, mice were first deeply anesthetized with isoflurane, then sacrificed by cervical dislocation under anesthesia, and blood and tissues were collected immediately.

### Labeling and tracing of MSC-sEV

To evaluate the tissue distribution of MSC-sEV, they were labeled with 1,1’-dioctadecyl-3,3,3’,3’-tetramethylindodicarbocyanine perchlorate (DiD; Invitrogen, USA) and injected into mice via tail vein. Six hours post-injection, mice were sacrificed, and organs (brain, heart, lungs, liver, spleen, and kidneys) were harvested. Imaging was performed using the IVIS SpectrumCT In Vivo Imaging System (PerkinElmer, USA). Additionally, kidney sections were stained with 4’,6-diamidino-2-phenylindole (DAPI) and imaged under a fluorescence microscope.

Additionally, in vitro observation of MSC-sEV uptake by RTECs was conducted using 3,3’-dioctadecyloxacarbocyanine perchlorates (DiO, Invitrogen, USA)-labeled MSC-sEV. DiO-MSC-sEV were added to the supernatants of HK-2 cells stimulated with PBS or LPS and incubated for 24 h. Uptake levels of MSC-sEV by RTECs under different conditions were assessed via confocal laser scanning microscopy (Leica, Germany), with quantitative analysis performed by flow cytometry.

### Percutaneous glomerular filtration rate measurement (GFR)

After depilating the renal region and inducing anesthesia with isoflurane, the GFR monitoring device (MediBeacon, Germany) was securely affixed to clean and dry skin. Mice were individually housed for 5 min post-anesthesia to ensure full consciousness. FITC-sinistrin tracer (7 mg/100 g body weight) was then administered via tail vein injection. Data were collected after 1 h of monitoring for subsequent analysis.

### Pathological staining

Renal tissue blocks fixed with 4% paraformaldehyde were paraffin-embedded and sectioned into 4-μm-thick slices. Sections were subsequently processed for hematoxylin-eosin (HE) staining, immunohistochemical staining, and immunofluorescence staining following standard protocols.

### Serological detection

Blood collected from mice was centrifuged at 3000 rpm for 10 minutes to isolate serum. Serum levels of creatinine, blood urea nitrogen (BUN), cystatin C, and lactate dehydrogenase (LDH) were quantified using microplate-based assays according to the manufacturer’s instructions (Nanjing Jiancheng Bioengineering Institute, China). Additionally, serum interleukin-1β (IL-1β) and interleukin-18 (IL-18) levels were determined by ELISA (Elabscience, China).

### mRNA sequencing

Total RNA was extracted from renal tissues of experimental groups using TRIzol Reagent (Invitrogen, USA). After quality verification with Agilent 2200 (Agilent Technologies, USA), qualified RNA samples were processed for cDNA library construction. Sequencing was performed on the DNBSEQ-T7 platform (MGI Tech, China) with 150-bp paired-end reads. Bioinformatics analyses included identification of differentially expressed genes (DEGs), Gene Ontology (GO) annotation, Kyoto Encyclopedia of Genes and Genomes (KEGG) pathway analysis, and Gene Set Enrichment Analysis (GSEA) based on sequencing reads.

### miRNA sequencing

Total RNA from MSC-sEV was extracted using the exoEasy Maxi Kit (QIAGEN, Germany). After quality validation, 50 ng of total RNA per sample was used to construct small RNA libraries. Sequencing was conducted on the Illumina NovaSeq 6000 platform (New England Biolabs, USA), with miRNA fragments sized 15-41 bp selected for analysis.

### Scanning electron microscopy (SEM)

Freshly isolated renal tissues were dissected into 1 mm³ blocks and fixed in 2.5% glutaraldehyde in electron microscopy fixative for 2 h. After three PBS washes, samples were post-fixed in 1% OsO₄ for 2 h, dehydrated through graded ethanol and acetone series, and subjected to critical point drying. Specimens were then coated with gold using an ion sputter coater (Hitachi, Japan) to enhance conductivity, and imaged under a scanning electron microscope (SEM; Hitachi, Japan).

### Dual-luciferase reporter assay

The 3′UTR luciferase reporter vector, miRNA/hNF-κB promoter-Luc and Renilla luciferase were co-transfected into HEK293T cells. Cells were lysed 48 h post-transfection, and luciferase activity in the lysates was measured using a Dual-Luciferase Assay Kit (HANBIO, China) with a microplate reader.

### HK-2 cells culture and intervention

The human renal tubular epithelial cell line HK-2 was purchased from the American Type Culture Collection (ATCC) and cultured in DMEM/F12 medium supplemented with 10% fetal bovine serum (FBS, Australia) and 1% penicillin-streptomycin. Cells were seeded in 6-well plates and cultured until reaching 80% confluence, followed by synchronization in serum-free DMEM/F12 medium for 12 hours. Subsequently, equal volumes of PBS or different doses of MSC-sEV (2 × 10⁸ particles or 1 × 10⁹ particles) were added to each well for pre-treatment. After 2 h, 50 μg/mL lipopolysaccharide (LPS; Sigma-Aldrich, L2880) was added, and cells were further cultured for 24 h before harvesting for subsequent experiments.

### Cellular oxidation and activity assay

Intracellular reactive oxygen species (ROS) levels were detected using the DCFH-DA fluorescent probe, while cell viability was assessed with propidium iodide (PI) staining, according to manufacturer instructions. Cells were cultured in 12-well plates and treated with designated agents as described above. Following 24-hour LPS stimulation, fluorescent probes were diluted 1:1000 and added to the culture. After 30-60 minutes of dark incubation, fluorescence microscopy imaging was performed.

### Flow cytometry

Cells were trypsinized, transferred to centrifuge tubes, and centrifuged at 1000 rpm for 5 min. The supernatant was discarded, and cell pellets were washed twice with PBS. Cells were then incubated with flow cytometry antibodies in the dark for 30 minutes. Subsequently, unbound antibodies were removed by centrifugation at 8000 × *g* for 5 min at 4 °C, followed by three PBS washes. The final pellet was resuspended in 500 μL PBS and analyzed using a flow cytometer (BD Biosciences, USA).

### Quantitative real-time PCR (qRT-PCR)

Total RNA from renal tissues, cells, and sEV was isolated using RNA extraction kits (Vazyme, China) following the manufacturer’s protocol. RNA concentration was measured by spectrophotometry, followed by reverse transcription to synthesize cDNA. qRT-PCR analysis was performed using ChamQ SYBR qPCR Master Mix (Vazyme, China). GAPDH and U6 served as internal controls for mRNA and miRNA, respectively. Relative expression levels were calculated via the 2^(-ΔΔCt)^ method. Primer sequences are listed in Table S1.

### Western blot

Total protein was extracted from samples using a high-efficiency lysis buffer (Biosharp, China). Protein concentrations were determined with a BCA protein assay kit (Beyotime Institute of Biotechnology, Shanghai, China). Proteins were separated on 4–20% SDS-PAGE gels and transferred onto 0.22 μm PVDF membranes (Millipore, USA). Membranes were blocked with 5% non-fat milk at room temperature for 1 hour, followed by overnight incubation with primary antibodies at 4 °C. Subsequently, membranes were incubated with horseradish peroxidase (HRP)-conjugated secondary antibodies (Santa Cruz Biotechnology, Dallas, TX, USA) in the dark at room temperature for 2 h. Protein signals were detected using an enhanced chemiluminescence detection system.

### Statistical analysis

Quantitative data were expressed as mean ± standard deviation (SD). Comparisons between two groups were performed using two-tailed unpaired Student’s *t* test. Comparisons among three or more groups were analyzed by one-way analysis of variance (ANOVA). Statistical analyses were conducted using SPSS 27.0.1. A *P* value < 0.05 was considered statistically significant.

## Supplementary information

Related Manuscript File

Supplementary Material

## Data Availability

The datasets used and analyzed during the current study are available from the corresponding author on reasonable request.
