# Supplementary material for: Mesenchymal stem cell-derived small extracellular vesicles suppress pyroptosis by delivering miR-125a-5p to improve acute kidney injury in sepsis
Source: Cell Death Discov. 2026 May 19;12:298. doi: 10.1038/s41420-026-03143-6 (PMC13357607; doi:10.1038/s41420-026-03143-6)
Supplement: Supplementary file 1 — Related Manuscript File [file 41420_2026_3143_MOESM1_ESM.docx]

**Mesenchymal stem cell-derived small extracellular vesicles suppress pyroptosis by delivering miR-125a-5p to improve acute kidney injury in sepsis**

Feng Chen^1,2*^, Tao-Tao Tang^1*^, Zhi-Qing Chen^1^, Qing Yang^1^, Yue Zhang^1^, Yi-Lin Zhang^1^, Jing Song^1^, Meng-Yun Wang^1^, Hong-Bin Yang^1^, Min Yang^3^, Suo-Fu Qin^3^, Zhe Guo^2^, Xue-Song Wang^2^, Zhong Wang^2#^, Lin-Li Lv^1#^, Bi-Cheng Liu^1#^

^1^ Department of Nephrology, Zhongda Hospital, Southeast University School of Medicine, Nanjing, China.

^2^ School of Clinical Medicine, Tsinghua University, Beijing, China.

^3^ Shenzhen Kexing Pharmaceutical Co., Ltd., Shenzhen, China.

# Corresponding authors.

E-mail addresses: liubc64@163.com (Bi-Cheng Liu), lvlinli@seu.edu.cn (Lin-Li Lv), wz523@mail.tsinghua.edu.cn (Zhong Wang)

* These authors contributed equally to this work.

**Supplementary Figures**


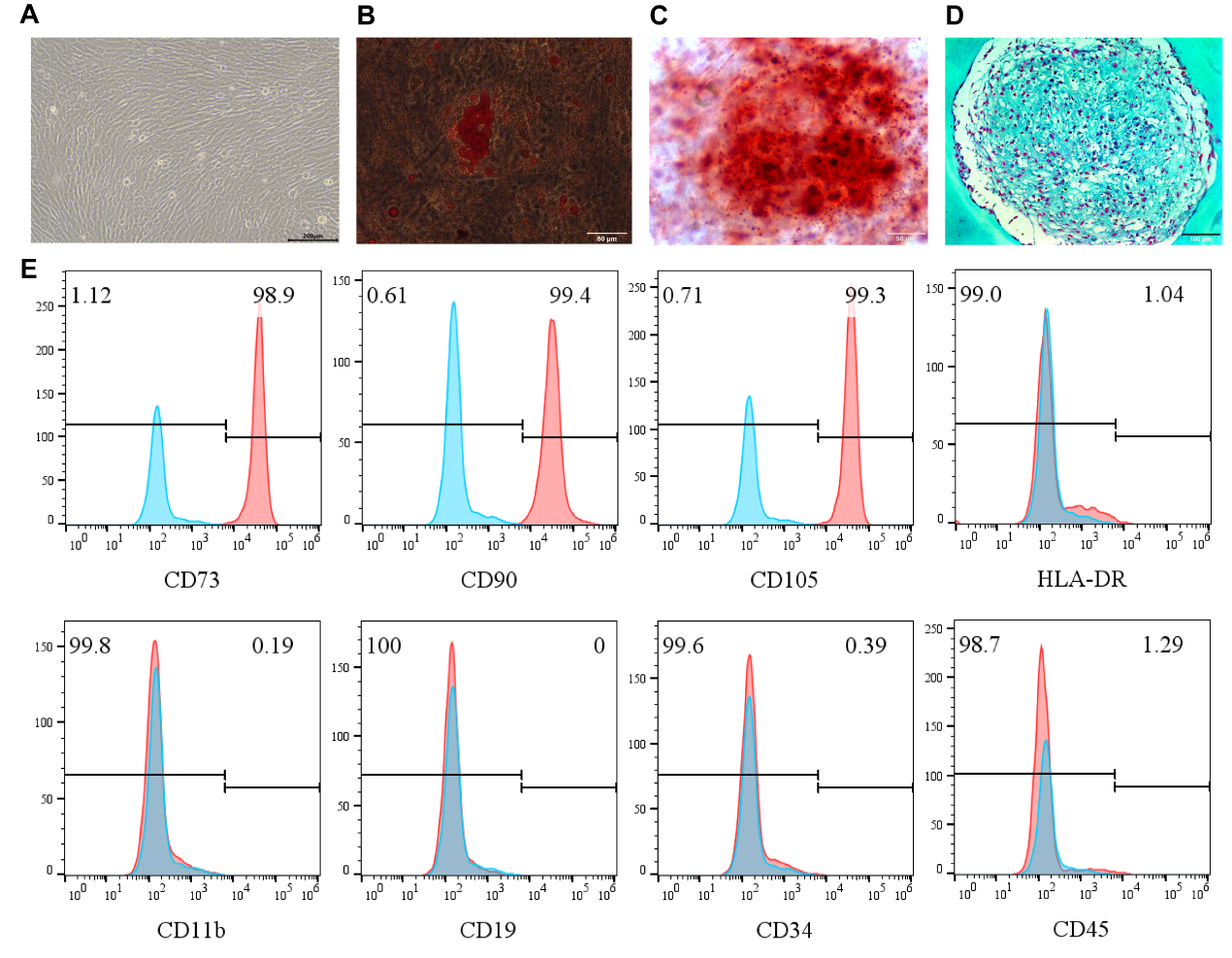


**Supplementary Figure S1. Characterization results of hucMSCs.** (A) Representative image of hucMSCs growth morphology. Scale bar, 200 μm. (B) Representative image of adipogenic differentiation of hucMSCs. Scale bar, 50 μm. (C) Representative image of osteogenic differentiation of hucMSCs. Scale bar, 50 μm. (D) Representative image of osteogenic differentiation of hucMSCs. Scale bar, 100 μm. (E) Flow cytometry identification of marker molecules for hucMSCs.


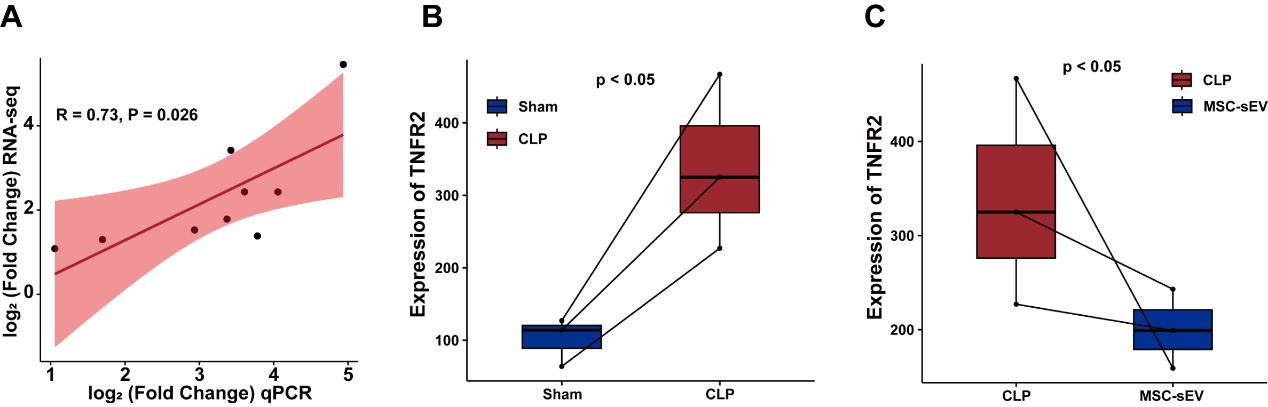


**Supplementary Figure S2. Expression of TNFR2 in the renal transcriptomes of mice.** (A) Correlation between transcriptomic profiling and qRT-PCR measurements. (B-C) Comparative TNFR2 expression in kidneys among Sham, CLP, and MSC-sEV groups.


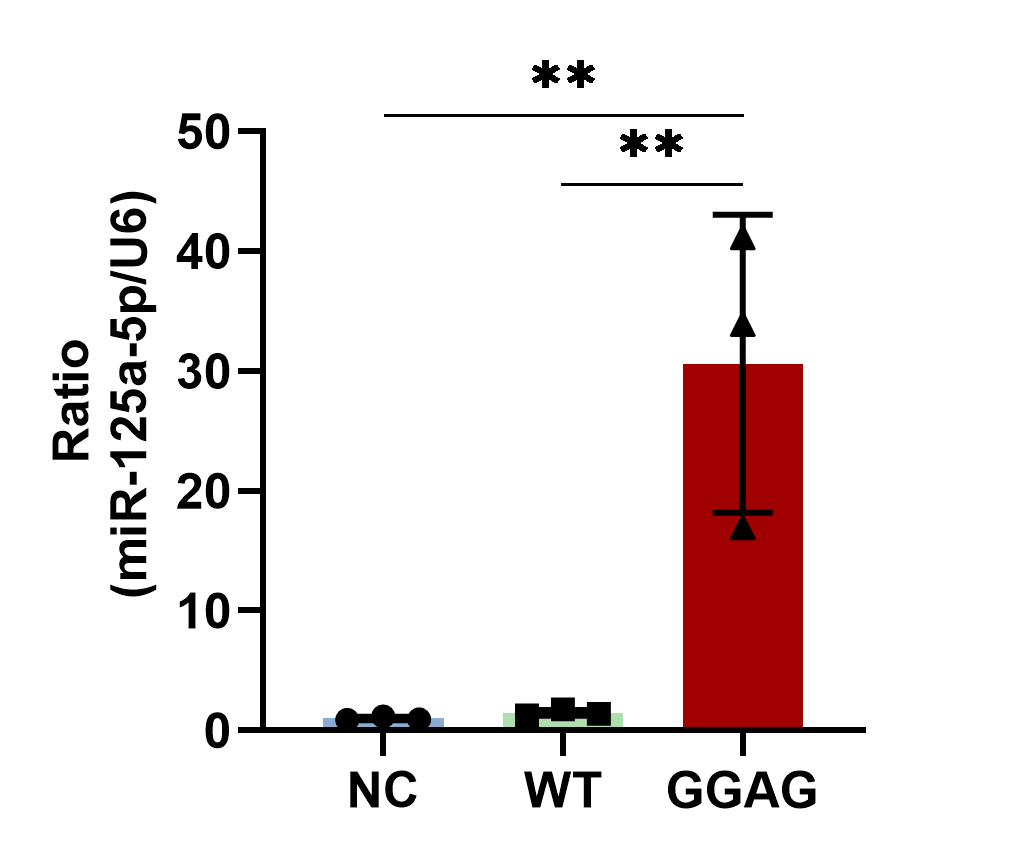


**Supplementary Figure S3. The relative content of miR-125a-5p in MSC-sEV following GGAG modification.** Using qRT-PCR quantification, we found that GGAG modification significantly increased the content of miR-125a-5p in sEVs by more than 20-fold compared to WT modification.

**Supplementary Table S1 Sequences of primers**

| **Gene** | **Forward (5’-3’)** | **Reverse (5’-3’)** |
| --- | --- | --- |
| Mus IL-1β | GAAATGCCACCTTTTGACAGTG | TGGATGCTCTCATCAGGACAG |
| Mus IL-6 | CTGCAAGAGACTTCCATCCAG | AGTGGTATAGACAGGTCTGTTGG |
| Mus IL-18 | CTTGGAGACATCCTGTCAGGG | AGTCACAAGACCAGGCATATTCT |
| Mus TNF-α | CTGAACTTCGGGGTGATCGG | GGCTTGTCACTCGAATTTTGAGA |
| Mus CCL2 | AGGTCCCTGTCATGCTTCT | GCTGCTGGTGATCCTCTT |
| Mus F4/80 | GCTGTAACCGGATGGCAAAC | CAGCAGGAAGGTGGCTATGG |
| Mus KIM-1 | TCAGAAGAGCAGTCGGTACAAC | TGTAGCTGTGGGCCTTGTAGT |
| Mus NGAL | TGGCCCTGAGTGTCATGTG | CTCTTGTAGCTCATAGATGGTGC |
| Mus TNFR2 | GTACTGCGCCTTGAAAACCC | CAGGATGCTACAGATGCGGT |
| Mus NLRP3 | CGTGTTGTCAGGATCTCGCA | GGTTGGTTTTGAGCACAGAGG |
| Mus Caspase-1 | TGCTGGAGGATCTGGGGTAT | GGCAGGCAGCAAATTCTTTCA |
| Mus GSDMD | GATCAAGGAGGTAAGCGGCA | CACTCCGGTTCTGGTTCTGG |
| Mus ASC | GCTGAGCAGCTGCAAAAGAT | GCAATGAGTGCTTGCCTGTG |
| Mus GAPDH | CTCTGCTCCTCCTGTTCGAC | GCGCCCAATACGACCAAATC |
| hsa-miR-125b-5p | CGCGTCCCTGAGACCCTAAC | / |
| hsa-miR-125a-5p | GCGTCCCTGAGACCCTTTAAC | / |
| U6 | TTCGGCAGCACATATACT | / |
| Homo IL-1β | ATGATGGCTTATTACAGTGGCAA | GTCGGAGATTCGTAGCTGGA |
| Homo IL-6 | ACTCACCTCTTCAGAACGAATTG | CCATCTTTGGAAGGTTCAGGTTG |
| Homo IL-18 | TTGACCAAGGAAATCGGCCT | CCATACCTCTAGGCTGGCTAT |
| Homo TNF-α | GAGGCCAAGCCCTGGTATG | CGGGCCGATTGATCTCAGC |
| Homo CCL2 | AGCAGCAAGTGTCCCAAAGA | GGTGTCTGGGGAAAGCTAGG |
| Homo GAPDH | GCATGGCCTTCCGTGTTC | GATGTCATCATACTTGGCAGGTTT |
