# Supplementary figures and images for: Mesenchymal stem cell-derived small extracellular vesicles suppress pyroptosis by delivering miR-125a-5p to improve acute kidney injury in sepsis
Source: Cell Death Discov. 2026 May 19;12:298. doi: 10.1038/s41420-026-03143-6 (PMC13357607; doi:10.1038/s41420-026-03143-6)

Original full length western blot.


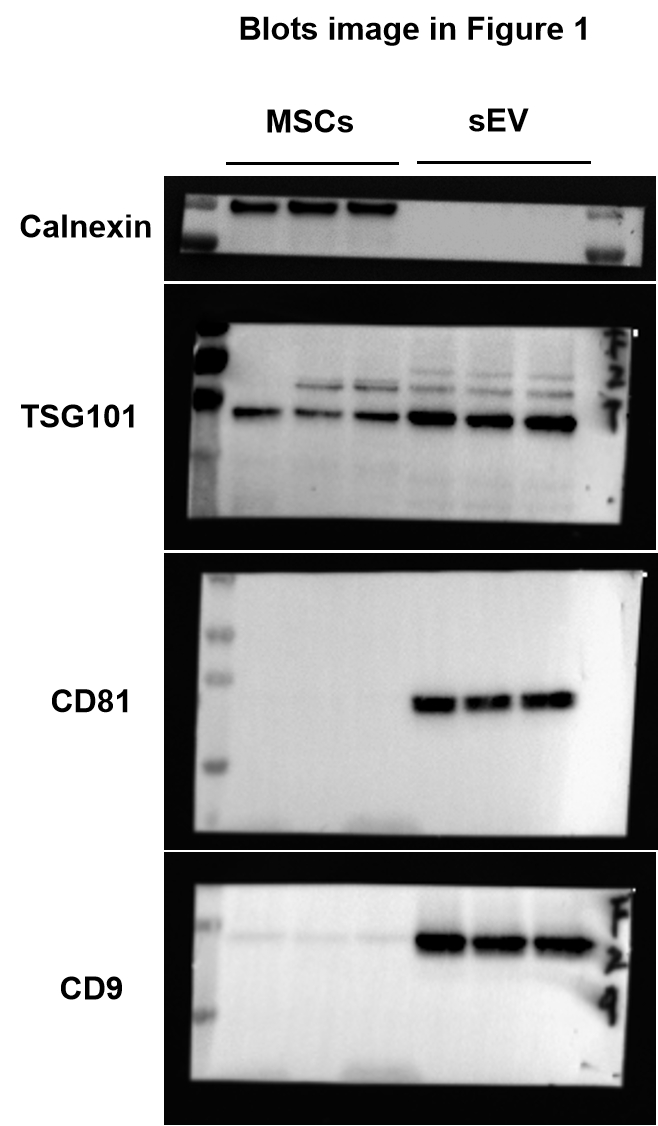


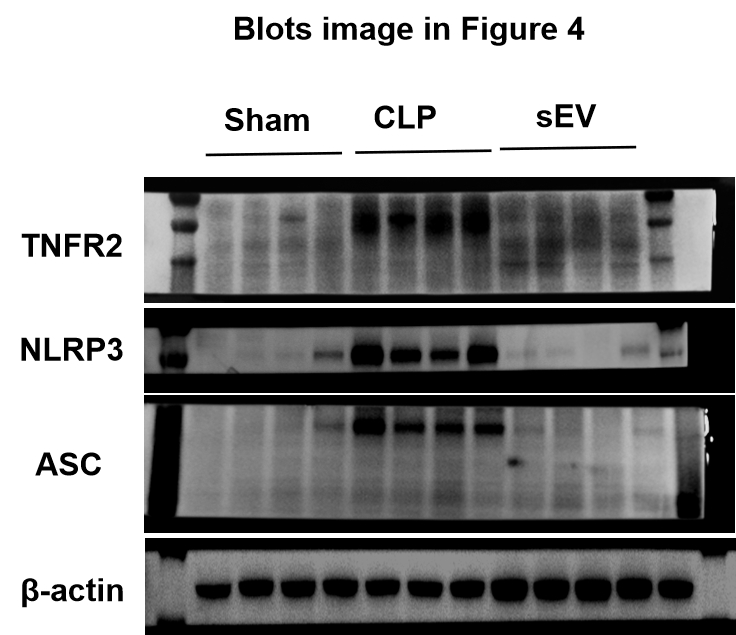


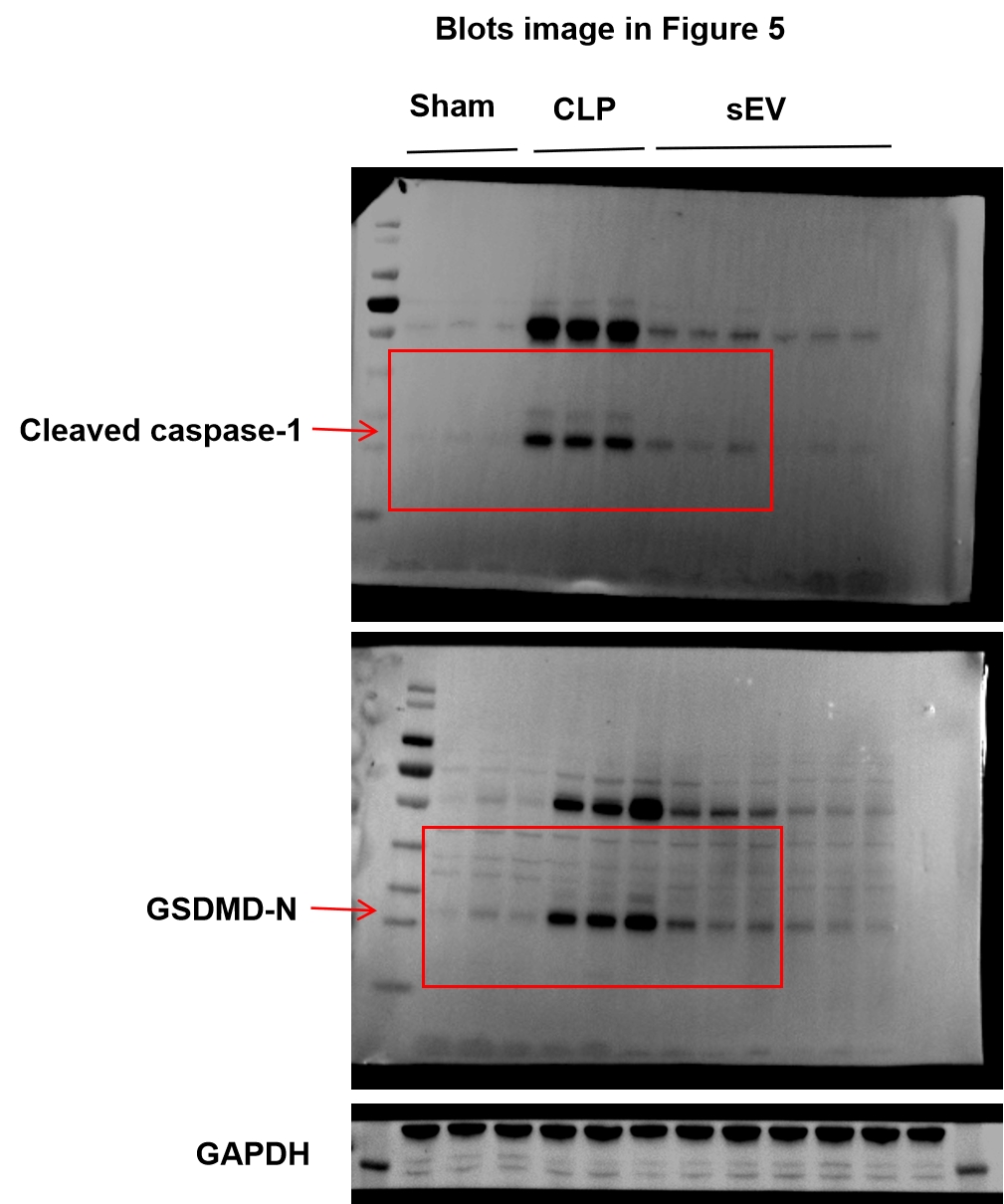


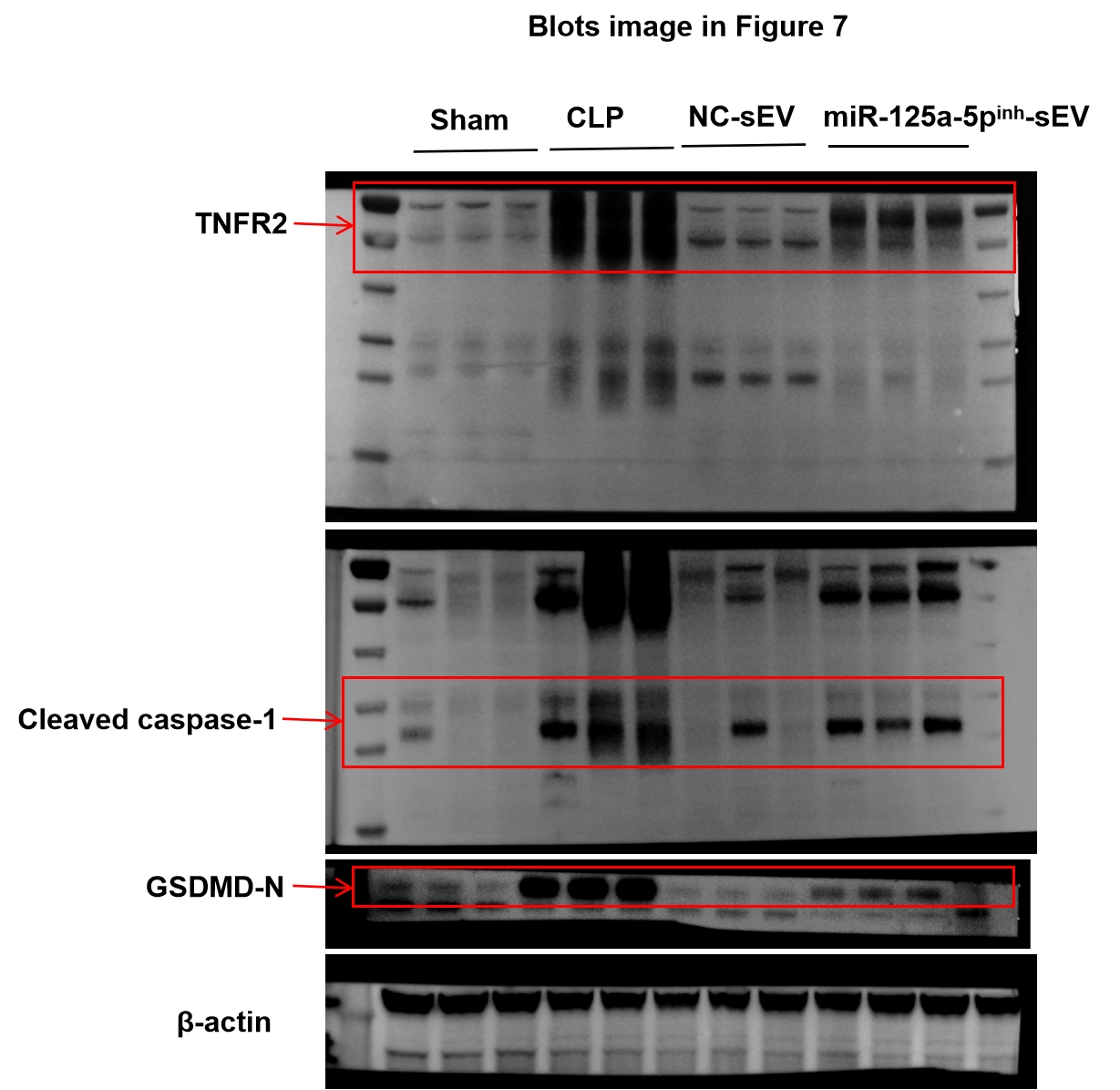


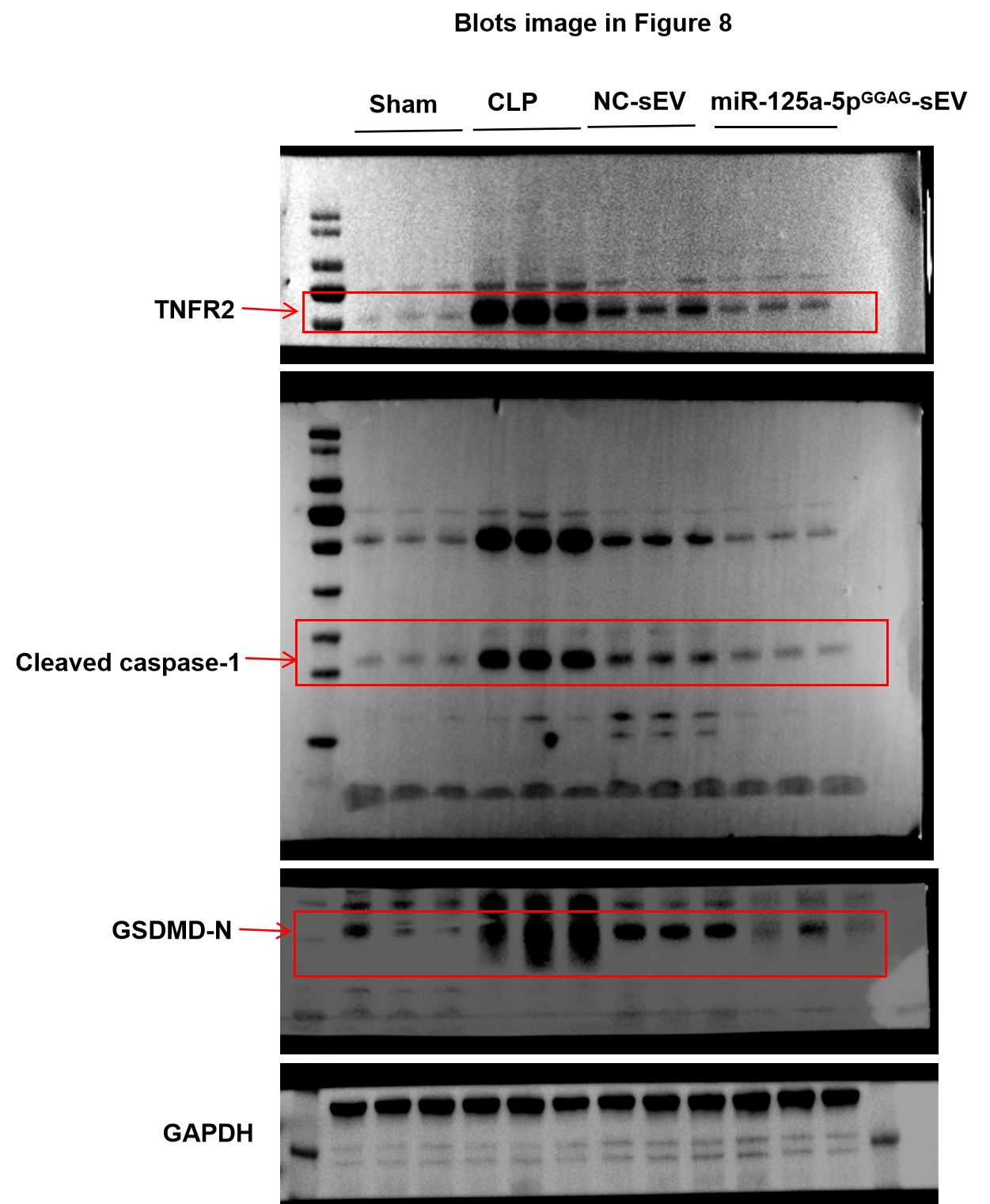

Supplement: Supplementary file 2 — Supplementary Material [file 41420_2026_3143_MOESM2_ESM.docx]
